# Manipulation of autophagy: a novelly potential therapeutic strategy for retinal neovascularization

**DOI:** 10.1186/s12886-018-0774-6

**Published:** 2018-04-27

**Authors:** Rong Li, Jin Tian, Junhui Du, Lei Zhao, Yang Yao, Zhaoxiang Yu, Weiping Chang, Rui Shi, Jing Li

**Affiliations:** 1 Department of Ophthalmology, the First Affiliated Hospital of Xi’an Medical University, Xi’an, Shaanxi China; 2 Department of Ophthalmology, the Weinan Central Hospital, Weinan, Shaanxi China; 3 Department of Ophthalmology, Xi’an Ninth Hospital Affiliated to Medical College of Xi’an Jiaotong University, Xi’an, Shaanxi China; 4 Department of Molecular Physiology and Biophysics, Holden Comprehensive Cancer Center, University of Iowa Carver College of Medicine, Iowa City, IA USA; 5 Department of Central laboratory, The First Affiliated Hospital of Xi’an Medical University, Xi’an, Shaanxi China; 6 Department of General surgery, The First Affiliated Hospital of Xi’an Medical University, Xi’an, Shaanxi China; 7 Department of Ophthalmology, Shaanxi Provincial People’s Hospital, Xi’an, Shaanxi China

**Keywords:** Autophagy, Angiogenesis, Retinal neovascularization, Hypoxia, VEGF

## Abstract

**Background:**

The relationship between the role of VEGF and autophagy in the process of retinal angiogenesis is still unclear. In this study, we explored this issue by using the mouse retinal vascular endothelial cell (RVEC) as a model.

**Methods:**

RVECs were divided into the following groups: control, hypoxia (H), 3-methyladenine (3-MA) + H, VEGF + H, 3-MA + VEGF+H, anti-VEGF antibody + H, 3-MA+ anti-VEGF antibody + H. We then examined activation of autophagy by detecting formation of autophagosomes with transmission electron microscopy (TEM) and by counting the number of green fluorescent protein-positive (GFP+) puncta in RVECs. The turnover of microtubule associated protein 1 light chain 3 B (LC3B) and VEGF were examined by western blot. Cell migratory capacity was measured with wound healing assay and transwell assay. The capillary formation assay was performed to investigate the angiogenic capacity.

**Results:**

Hypoxia led to an increased number of autophagosome and of the GFP+ puncta, an increased ratio of LC3B-II/I and enhanced migratory and capillary-formation capacities of RVECs. Pre-treatment with 3-MA attenuated activation of autophagy and abrogated the enhanced cell migration and capillary formation under hypoxia. Exposure to VEGF significantly increased migratory and capillary formation capacities of RVECs under hypoxia and 3-MA decreased VEGF-induced angiogenesis without its expression. Formation of autophagosome, the number of GFP+ puncta of RVECs and expression of LC3B-II/I were both elevated in cells treated with anti-VEGF antibody and these effects were partially inhibited by 3-MA pretreatment.

**Conclusion:**

Our present data may identify autophagic response as a novel target for enhancing the therapeutic efficacy of angiogenesis inhibitors.

**Electronic supplementary material:**

The online version of this article (10.1186/s12886-018-0774-6) contains supplementary material, which is available to authorized users.

## Background

Hypoxia can prompt release of angiogenic growth factors to stimulate proliferation and differentiation of vascular endothelial cells and induce the process of angiogenesis [1, 2]. The retina, as one of the most metabolically active human tissues, is highly sensitive to hypoxia and consequent oxidative stress. In recent years, a large body of studies in the mechanisms of angiogenesis and in the development of therapeutic strategies targeting retinal neovascularization have been carried out. The development of anti-VEGF drugs has revolutionized the treatment of retinal neovascularization as VEGF is one of the critical angiogenic factors [3]. However, the underlying mechanisms for retinal neovascularization still remain largely unclear and up to 30% of patients have no reaction to the anti-VEGF drugs [4]. Therefore, there is an urgent need to explore the mechanisms of retinal angiogenesis and develop other effective therapeutic strategies.

Autophagy is a complex, multistep process to degrade intracellular components through forming autophagosomes, which then fuse with lysosomes to form autolysosomes, and it is of fundamental importance in maintaining cell homeostasis [5]. Autophagy has been reported to play important roles in development and tissue remodeling and been involved in multiple pathological processes. Therefore, its roles in cell proliferation, death and other cellular functions have become a hot topic in research in recent years [6–8]. Autophagy is a double-edged sword in the development of retinopathies in retinal cells under oxidative stress: altered autophagy may have a neuroprotective effect or contribute to photoreceptor degeneration via initiating cell apoptosis [9, 10]. However, the exact roles of autophagy in angiogenesis of ocular vascular endothelial cells have not been fully elicited.

Our previous study suggested that activation of autophagy by CoCl_2_-induced hypoxia could promote angiogenesis of RF/6A, a rhesus macaque choroid-retinal endothelial cell line, and that these effects were effectively inhibited by blocking autophagy [11]. However, despite the wide use of CoCl_2_ in mimicking cellular hypoxia, it is difficult to distinguish whether these effects were caused by hypoxia or by the potential effects of CoCl_2_. In addition, the RF/6A cell line is a spontaneously transformed endothelial cell line of mixed origin (choroidal and retinal), and these two types of cells exhibit molecular diversity and respond differently to external stimuli. Therefore, retinal endothelial cell from a single origin is a better model for investigating the role of autophagy in retinal angiogenesis. Furthermore, whether autophagy activation is associated with VEGF-induced angiogenesis and whether the effect of anti-VEGF agents can be affected by autophagy activation are still unclear. Given this, in this study we investigated the role of autophagy in retinal angiogenesis and the relationship between autophagy activation and VEGF in the process of angiogenesis by using mouse retinal vascular endothelial cell (RVECs) under physical hypoxia in vitro.

## Methods

### Cell culture

Primary mouse RVECs were obtained from the retina of C57BL/6 J mice provided by the experimental animal center of Xi’an Medical University following conventional protocols [12, 13]. Mice were euthanized by an overdose of intraperitoneal sodium pentobarbital and their eyes were enucleated. The eyeballs of mice were obtained and the residual retinal pigment epithelial cells and the visible main blood vessels in retinal tissues were removed. The separated retinal tissue was placed into a new culture dish and minced as finely as possible. The minced retinal tissues were then digested with collagenase and the digestion was terminated until cells were separated under an inverted phase contrast microscope (IX51, Olympus, Japan). The dissociated cells were then filtered through a 100 μm cell strainer and spin at 1000 rpm for 5 min. The cell pellets were resuspended and incubated in complete culture medium for mouse retinal microvascular endothelial cells (CM-M114, Procell, China). Microvascular endothelial cells migrated from vascular fragments and grew into clones in a cobblestone shape. When visible clones were formed on the plate, they were marked by labeling on the bottom of the dish. Cells other than the clones were scratched off using a cell scraper. The remaining clones were then picked and cultured at 37 °C in a humidified 5% CO_2_ atmosphere. When reached a confluence of 70–80%, RVECs were harvested by digestion with 0.25% trypsin and passaged in a ratio of 1:3 to expand the cells. In this study, 150 mice were used and all experiments were performed based on the second passage of cells after separation.

### Immunofluorescence

For immunofluorescent analysis of CD34 expression, cells were grown on collagen I-coated coverslips placed on 6 well plates and reached a confluence of 60%–70% before the assay. Cells were fixed on coverslips with 4% paraformaldehyde in PBS for 15 min at room temperature (RT) and then permeabilized with 0.5% Triton X-100 for 20 min. After blockage in normal goat serum for 30 min at RT, cells were stained with primary CD34 antibody (1:200, Abcam, USA) at 4 °C for overnight in a wet box and then incubated with Cy3-conjugated goat anti-rabbit secondary antibody (1:100, Wuhan Boster, China) for 1 h at RT in dark. In the control cells, primary antibody was replaced with a mouse IgG isotype control (1:100, Sigma, USA). Coverslips were mounted to slides with ProLong Gold Antifade Mountant with DAPI (Thermo Fisher Scientific, USA). Images were collected and analyzed using a fluorescence microscope (Olympus BX53, Olympus, Japan).

### Cell grouping and treatments

Firstly, RVECs were randomly divided into three groups to investigate autophagy under hypoxia. 1 ml of RVECs suspension at 5 × 10^5^ cells/ml was seeded on each well of six-well culture plates in serum-free medium. For the hypoxic group, cells were kept in a hypoxic incubator for 24 h (1% O_2_/5%CO_2_/94%N_2_, BioSpherix, USA) as previously described [14]. For the 3-MA + hypoxia group, cells were pre-treated with 5 mM 3-MA (Sigma, USA), an autophagy inhibitor, for 4 h and then incubated under hypoxic condition for 24 h. Parallel cultures kept in a normoxic incubator served as the control group. Secondly, to investigate the relationship of autophagy and VEGF in angiogenesis of RVECs, the cells were pre-treated with 3-MA of 5 mM for 4 h or/and VEGF-A (Peprotech, USA) of 20 ng/ml for 4 h. To investigate the mutual effects of autophagy and anti-VEGF, the cells were pre-treated with 5 mM 3-MA for 4 h or/and an anti-VEGF-A neutralizing antibody ((RD, USA) of 10 μg/ml for 1 h. Then, the RVECs in the latter four groups were incubated in a sealed, hypoxic incubator for 24 h.

### Transmission electron microscopy (TEM)

Formation of autophagosome was detected by TEM. Briefly, after treated for indicated time, cells in different groups were harvested and fixed with 2.5% glutaraldehyde in 0.1 M phosphate buffer at 4 °C for 2 h. After washed with PBS for 3 times, cells were post-fixed with 1% osmic acid at 20 °C for 2 h. Cells were then dehydrated by a serial gradient ethanol and then embedded in Embed-812 medium for 48 h. The blocks were cut into ultra thin sections of 60–80 nm using an Ultrotome (Leica, Germany) on uncoated copper grids, which were stained with 0.2% lead citrate/1% uranyl acetate. Images were recorded under a TEM (HT7700-SS, HITACHI, Japan) according to manufacturer’s protocol.

### GFP- LC3B plasmid construction and transfection

The full-length sequence encoding for mouse LC3B was amplified from a PUC19-mus-LC3b plasmid by PCR with the following primers: Forward GGACTCAGATCTCGAGATGCCGTCCGAGAAGACCTT, Reverse: TGGTGGCGATGGATCCCACAGCCATTGCTGTCCCGA. The purified PCR product was then inserted into a pEGFP-N3 vector between the XhoI and BamHI sites (referred as pEGFP-LC3B). To transiently overexpress LC3B, 1 μg of the pEGFP-LC3B plasmid was transfected into the RVECs using lipofectamine 2000 (Thermo Fisher Scientific, USA) according to the manufactures instructions. The cells transfected with 1 μg of the empty pEGFP-N3 vector were used as a control. About 20 h post transfection, RVECs were treated with either vehicle or 3-MA for 4 h followed by hypoxia for 24 h. GFP-positive cells and cells with GFP+ puncta were counted under the fluorescence microscope. The percentage of cells with GFP+ puncta over total GFP-positive cells was counted among 150 cells in 3 repeats.

### Western blot analysis

The expression of LC3B-I/II and VEGF were analyzed by western blot as previously described [15]. REVCs were treated as mentioned above. Cells were lysed in the RIPA buffer (Beyotime, China), and protein concentration was measured by using a BCA Protein Assay kit (Beyotime, China). Totally 40 μg cell lysate was separated by 10% sodium dodecyl sulfate polyacrylamide gel electrophoresis (SDS-PAGE) at 120 V. The proteins were then transferred to a polyvinylidene fluoride (PVDF) membrane (Millipore, USA) at 200 mA for 90 min. The membranes were blocked with 5% non-fat dried milk in TBST for 1 h at room temperature and then incubated with LC3B antibody (1:1000, Cell Signaling, USA), VEGF-A antibody(1:1000, abcam, USA) and GADPH rabbit pAb (1:1000, Hangzhou Xianzhi biology co., LTD, China) primary antibodies for overnight at 4 °C, followed by incubation with horseradish peroxidase (HRP)-conjugated goat anti-rabbit secondary antibody (1:50000, Boster, China) for 1 h at RT. Then the membranes were incubated with ECL Western Blotting Substrate (Thermo Fisher Scientific, USA) and imaged using a ChemiDoc XRS imaging system (Bio-Rad, USA). The gray scale intensity of bands was analyzed using the QuantityOne software (Bio-Rad, USA) and the signals of LC3B I/II and VEGF were normalized to that of GAPDH.

### Wound healing assay

RVECs were seeded into 6 well plates at a density of 5 × 10^5^/well and treated as above-mentioned. The confluent monolayer of RVECs was then wounded with a p200 pipette tip, washed with PBS for 3 times and cultured in serum free M199 medium for 24 h at 37 °C in a 5% CO_2_ incubator. The migration area of cells that had migrated from the edge of the wound was recorded by using an inverted phase contrast microscope and calculated using Image J software (NIH, USA). The original wound area at 0 h was used as a baseline for comparing the migratory area of cells under different treatments. Wound healing rate = [(wound area at 0 h- wound area at 24 h) / wound area at 0 h] × 100%.

### Transwell assay

Cells were harvested by trypsinization and resuspended in serum free M199 medium at 2 × 10^4^cell/ml. Then 200 μl cell suspension were seeded in the upper part of the transwell inserts (8 μm pore membranes, Costar, USA) in a 24-well plate with 800 μl 10% FBS supplemented M199 medium placed in the lower compartment. After incubation for 24 h, cells on the upper side of the membrane were wiped off by using a cotton bar and cells that passed through the membrane were fixed on the membrane using 10% ethanol for 30 min and then stained with 5% crystal violet for 10 min. Five randomly selected fields were imaged per insert and the number of cells that passed through the membrane was counted under a phase contrast microscope.

### Endothelial cell capillary formation assay

The capillary formation capacity of RVECs was assayed by using an in vitro capillary formation assay as previously described [9]. RVECs were seeded into 6 well plates at a density of 5 × 10^5^ cells/well and treated as mentioned above. For the capillary formation assay, 24 well plates were pre-coated with 200 μl Matrigel (BD Biosciences, USA). The treated RVECs were then seeded in the wells at a density of 2 × 10^5^ cells/well and incubated at 37 °C for 8 h. Five randomly chosen fields of the Matrigel were imaged under a microscope at× 200 magnification. The number of branching points was determined using the Image J software (NIH, USA). Cell clusters with at least three tubular structures emanating out were considered to be a branching point.

### Statistics

Data were analyzed using SPSS 19.0 software (IBM, USA). All values were presented as mean ± SD of at least 3 experiments. The means of different treatment groups were compared using the one-way analysis of variance (ANOVA) and the LSD post hoc test. Two-sided *P*-values were calculated and *P* < 0.05 was considered as significantly different.

## Results

### Culture and identification of mouse RVECs

**Fig. 1:**
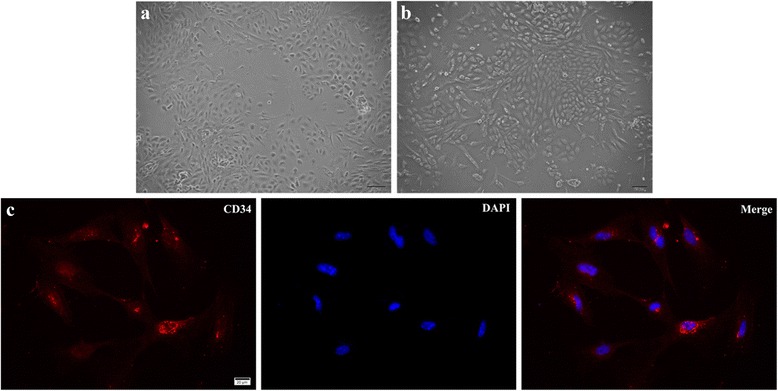
Identification of mouse RVECs. **a** Morphology of short-spindle like mouse RVECs at an early stage. **b** Images of cells with a cobblestone shape. **c** Representative images of primary mouse RVECs by immunofluorescence (Red: CD34, located mainly in the cytoplasm; Blue: DAPI; Bar = 50 μm)

Mouse retinal microvascular fragments were seeded and adherence of the primarily cultured RVECs was observed within 24 h and significantly increased within 36 h. Under microscope, the RVECs migrated from vascular fragments and grew into clones. Cells were short-spindle like at an early stage and gradually grew into monolayer with a cobblestone shape (Fig. 1a and b). Expression of CD34 was observed mainly in the cytoplasm of most cells by immunofluorescence (Fig. 1c). In together, the cell morphology and CD34 status confirmed that the cultured cells were RVECs.

### Autophagy activation in RVECs under hypoxia

**Fig. 2:**
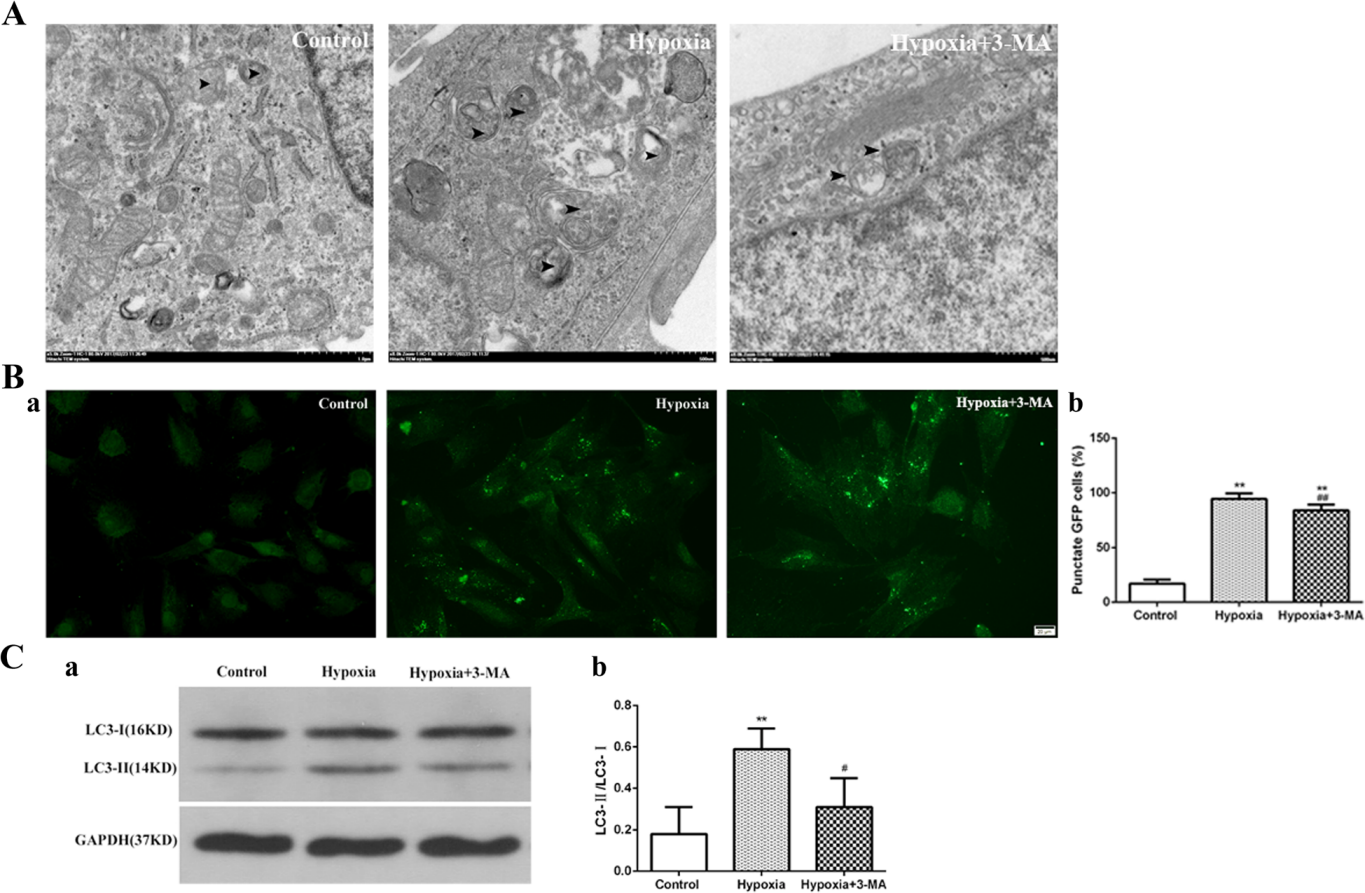
Autophagy was activated in RVECs under hypoxia. **a** Representative TEM images of autophagosome formation in RVECs (The arrows indicate the double-membrane bounded vacuoles digesting organelles or cytosolic contents, Bar = 20 μm). **b** Representative images of endogenous GFP+ puncta visualized by fluorescence microscopy (Bar = 20 μm) (a) and statistical histogram of percentage of punctate GFP cells in different groups (b). **c** Immunoblot of LC3B-I and LC3B-II in RVECs in the three groups (a). The histogram is the quantification of 3 independent experiments (b). (** *P*<0.01 VS Control; # *P*<0.05 VS Hypoxia; ## *P*<0.01 VS Hypoxia)

We firstly monitored the activation of autophagy in RVECs under different conditions by TEM. The results indicated that formation of autophagic vacuoles was significantly increased in the hypoxia group compared with the normoxia group and the effects of hypoxia on autophagic vacuole formation was blocked by pre-treatment with 3-MA (Fig. 2a). We then counted the number of autophagosome in cells ectopically expressing a GFP-LC3B chemiatric protein via visualizing the GFP+ puncta that represent autophagosomes under a fluorescence microscope. As shown in Fig. 2b, in the hypoxia group, the number of GFP+ puncta was significantly higher than that of the control group (94.44 ± 4.96 vs. 17.11 ± 3.52, *P* < 0.01), and the number of GFP+ puncta was decreased in the 3-MA plus hypoxia group compared to the hypoxia group (83.98 ± 5.39 vs. 94.44 ± 4.96, *P* < 0.01) (Additional file 1). To further observe the activation of autophagy in RVECs, we measured LC3B turnover by western blot [16]. In cells growing under normoxic condition the ratio of LC3B-II/I was significantly lower than that of the hypoxia group (0.18 ± 0.13 vs. 0.59 ± 0.10, *P* < 0.01), while pre-treatment with 3-MA significantly decreased the LC3B-II/I ratio in RVECs exposed to hypoxia (0.31 ± 0.14 vs. 0.59 ± 0.10, *P* < 0.05) (Fig. 2c) (Additional file 2). In together, these data suggested that low oxygen tension was a potent inducer of autophagy in RVECs under hypoxic conditions and that induction of autophagy by hypoxia could be partially blocked by 3-MA.

### Cell migration was enhanced in RVECs under hypoxia

**Fig. 3:**
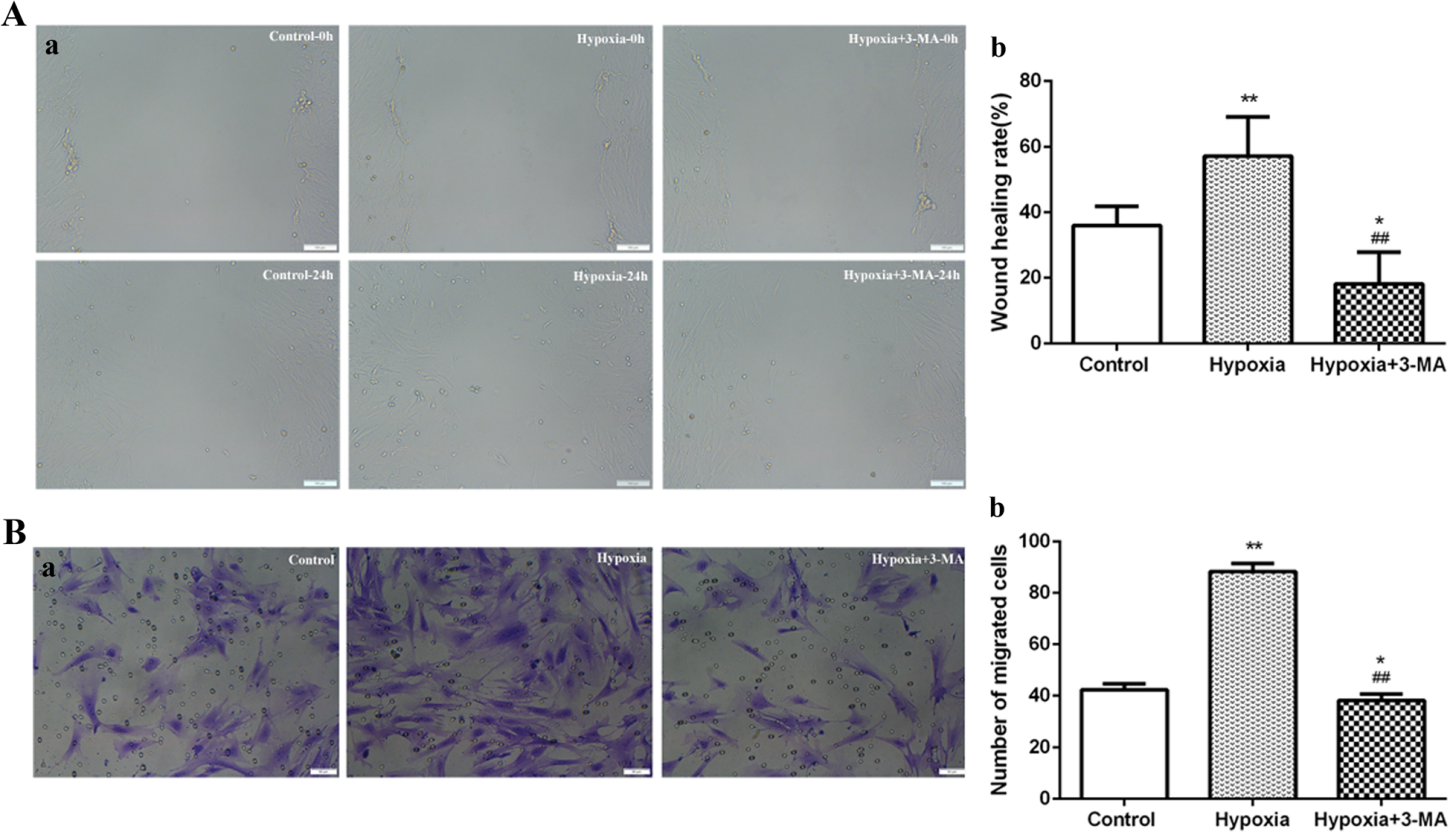
Cell migration was enhanced in RVECs under hypoxia. **a** Representative images of the wound healing assay for cell migration (Bar = 100 μm) (a) and the histogram represents the quantification of wound healing rate in 3 independent experiments (b). (* *P*<0.05 VS Control;** *P*<0.01 VS Control;## *P*<0.01 VS Hypoxia). **b** Representative images of transwell assays for the migration of RVECs exposed to hypoxia (a). The numbers of migrated cells were calculated based on 3 independent experiments (b). (* *P*<0.05 VS Control;** *P*<0.01 VS Control;## *P*<0.01 VS Hypoxia)

The wound healing assay demonstrated that exposure to hypoxia significantly increased cell migratory capacity at 24 h in RVECs compared with control cells under normoxia (57.26% ± 11.98% vs. 36.02% ±5.84%, *P* < 0.01), and that 3-MA significantly attenuated the effect of hypoxia on cell migration (18.16% ± 9.73% vs. 57.26% ± 11.98%, *P* < 0.01) (Fig. 3a) (Additional file 3). Moreover, the transwell assay showed similar results as the wound healing assay. The number of migrated cells in the hypoxia group was significantly higher than that of the controls cells (88.40 ± 3.05 vs. 42.40 ± 2.30, *P* < 0.01). However, the migrated cells of the 3-MA plus hypoxia group was obviously lower than that of the hypoxia group (38.20 ± 2.39 vs. 88.40 ± 3.05, *P* < 0.01) (Fig. 3b) (Additional file 3). These results suggested that autophagy might be involved in the enhanced cell migratory capacity induced by hypoxia.

### Capillary formation of RVECs was increased under hypoxia

**Fig. 4:**
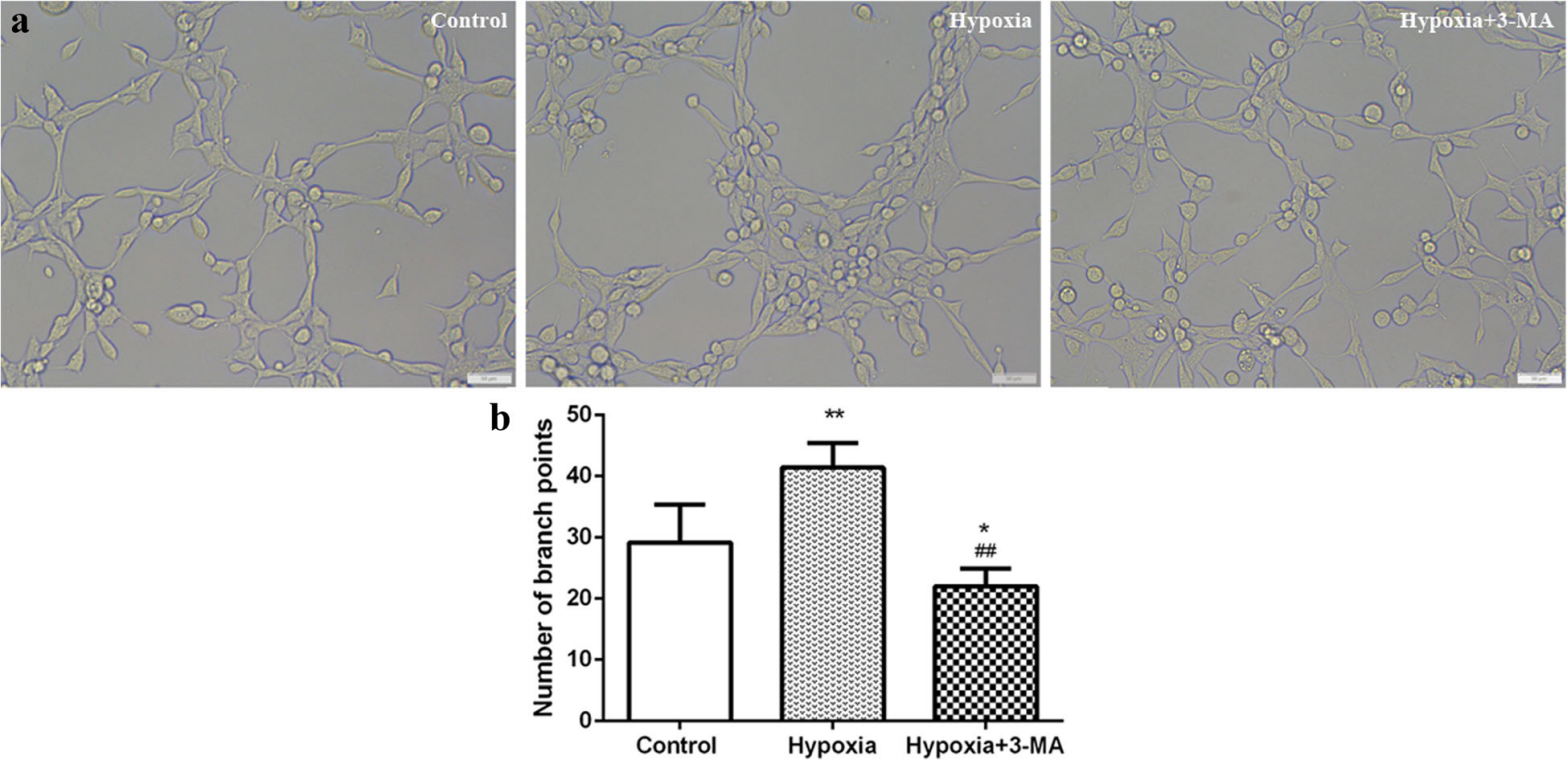
Increased capillary formation capacity of RVECs under hypoxia. **a** Representative images of capillary formation of RVEC on Matrigl (Bar = 50 μm). **b** Quantification of the number of branch points in different groups. The histograms represent results of 3 independent experiments (* *P*<0.05 VS Control;** *P*<0.01 VS Control;## *P*<0.01 VS Hypoxia)

The capillary formation assay demonstrated that hypoxia significantly increased the number of branching points at 24 h in RVECs compared with control cells under normoxia (41.40 ± 4.04 vs. 29.20 ± 6.10, *P* < 0.01) and that blockade of autophagy by 3-MA significantly inhibited the effect of hypoxia (22.00 ± 2.92 vs. 41.40 ± 4.04, *P* < 0.01) (Fig. 4a and b) (Additional file 3), suggesting that autophagy might account for the increase in capillary formation capacity of RVECs under hypoxia.

### VEGF-induced cell migration of RVECs was attenuated by autophagy inhibition

**Fig. 5:**
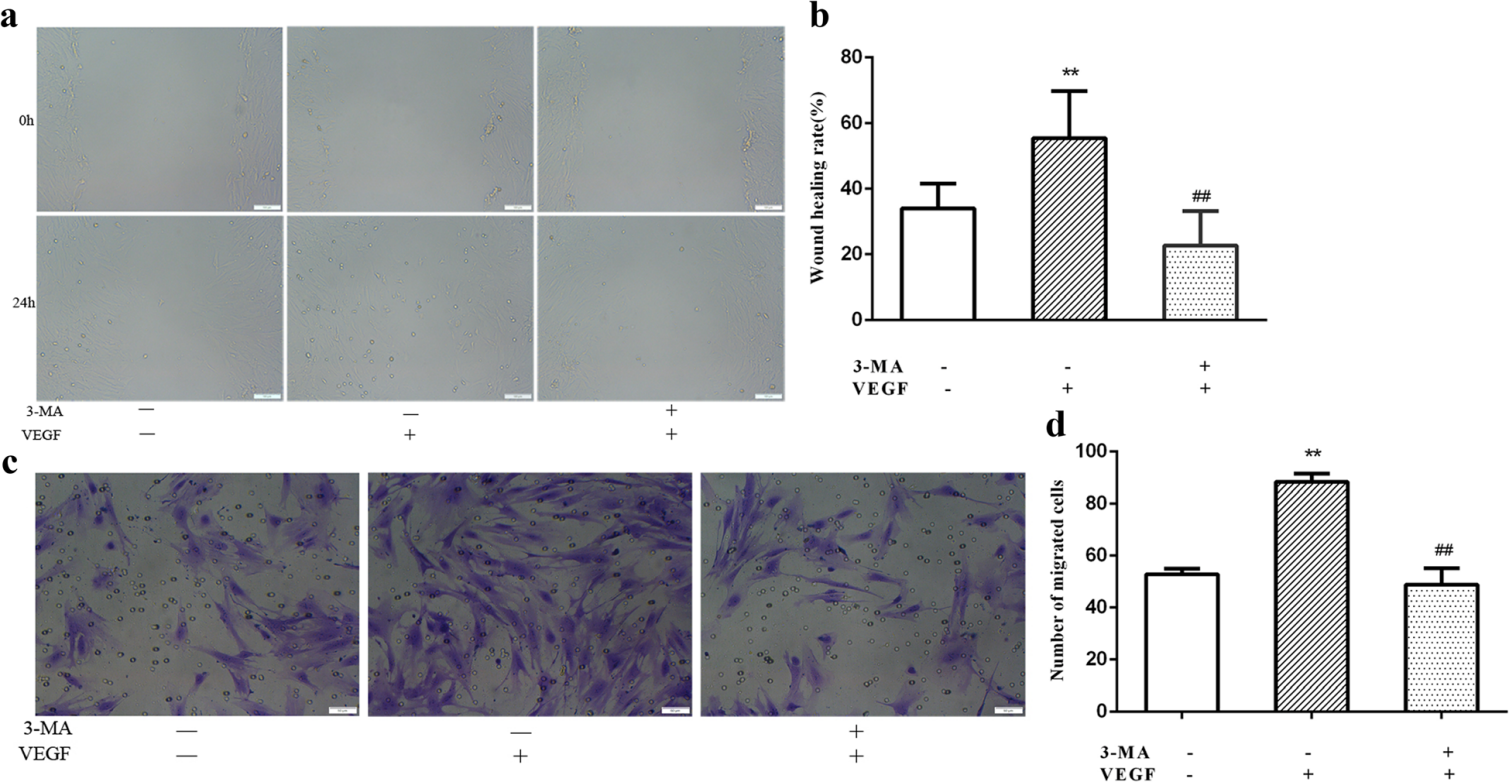
Representative images of cell migration by wound healing assay for the three groups at 0 h and 24 (**a**) (Bar = 100 μm) and statistical histogram of wound healing rate in different groups. Cells without 3-MA treatment and VEGF were used as basal controls (**b**) (** *P*<0.01 VS Control;## *P*<0.01 VS cells with VEGF treatment). Representative images of cell migration by transwell assay for the three groups (**c**) (Bar = 50 μm) and statistical histogram of number of migrated cells in different groups (**d**) (** *P*<0.01 VS Control without 3-MA and VEGF treatment;## *P*<0.01 VS cells with VEGF treatment)

The wound healing assay demonstrated that exposure to VEGF significantly increased cell migratory capacity presented as the wound healing rate at 24 h in RVECs compared with control cells under hypoxia (55.41% ± 14.36% vs. 34.00% ±7.58%, *P* < 0.01), and that 3-MA significantly attenuated the effect of VEGF on cell migration of RVECs (22.65% ± 10.51% vs. 55.41% ± 14.36%, *P* < 0.01) (Fig. 5a, b) (Additional file 3). Moreover, the transwell assay showed similar results as the wound healing assay. The number of migrated cells with VEGF in the culture medium was significantly higher than that of the cells without VEGF treatment (88.40 ± 3.05 vs. 52.80 ± 2.17, *P* < 0.01). However, the migrated cells of the group with 3-MA plus VEGF was obviously lower than that of the group with only VEGF (48.80 ± 6.34 vs. 88.40 ± 3.05, *P* < 0.01) (Fig. 5c, d) (Additional file 3). These results suggested that inhibition of autophagy may decrease VEGF-induced cell migration of RVECs under hypoxic condition.

### VEGF-induced tube formation of RVECs was attenuated by autophagy inhibition

**Fig. 6:**
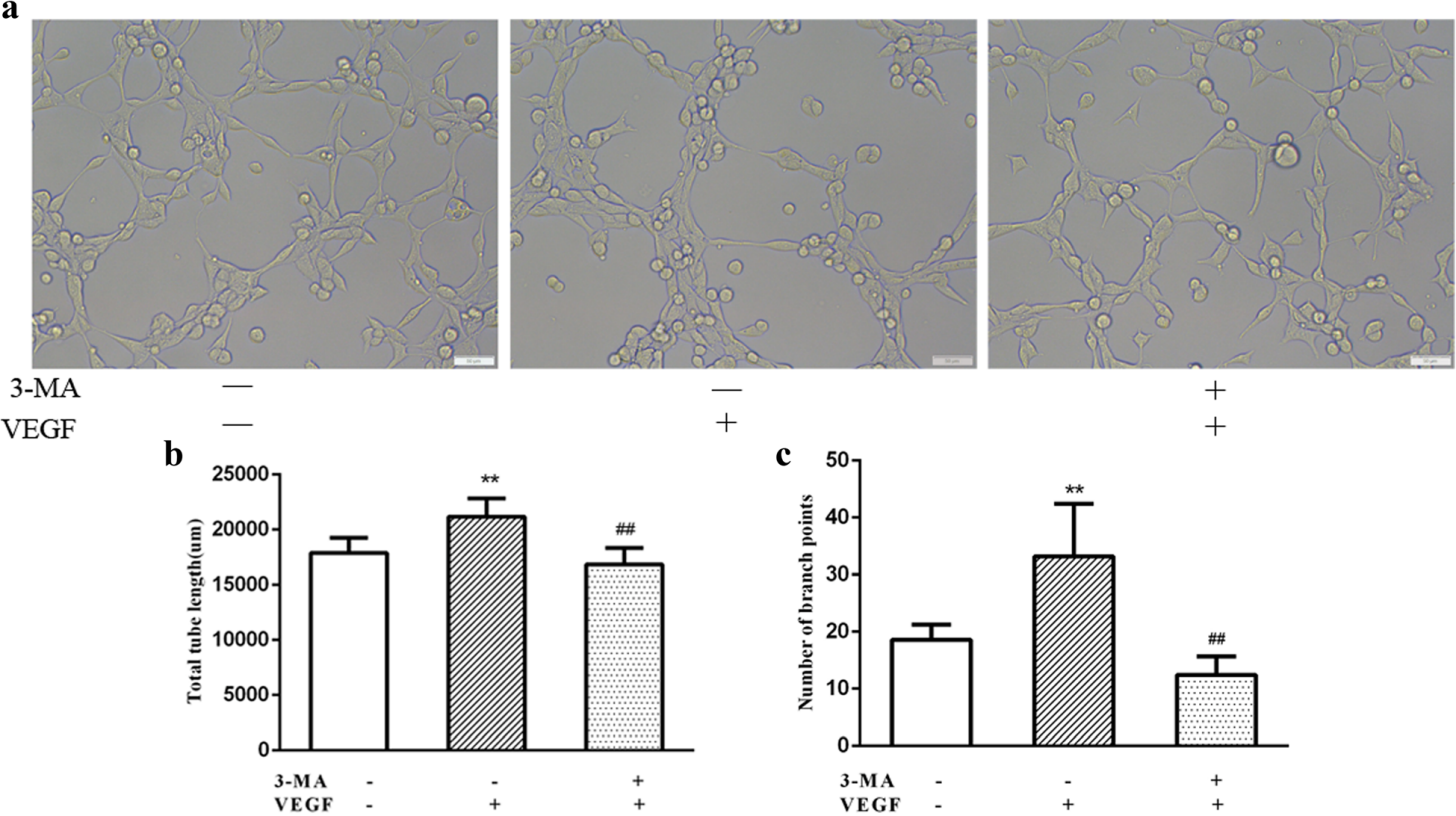
Representative images of tube formation of RVECs on Matrigl (**a**) (Bar = 50 μm) and statistical histogram of total tube length (**b**) and the number of branch points in different groups (**c**) (** *P*<0.01 VS Control without 3-MA and VEGF treatment;## *P*<0.01 VS cells with VEGF treatment)

The capillary formation assay demonstrated that exposure to VEGF treatment significantly increased the total tube length as well as the number of branch points at 24 h in RVECs compared with control cells under hypoxia (21,173.40 ± 1678.78 μm vs. 17,889.80 ± 1340.03 μm, *P* < 0.01; 33.20 ± 9.23 vs. 18.60 ± 2.61, *P* < 0.01; respectively) and blockade of autophagy by 3-MA significantly inhibited the effect of VEGF (16,853.80 ± 1476.55 μm vs. 21,173.40 ± 1678.78 μm, *P* < 0.01; 12.40 ± 3.29 vs. 33.20 ± 9.23, *P* < 0.01; respectively) (Fig. 6) (Additional file 3), suggesting that inhibition of autophagy may decrease VEGF-induced tube formation of RVECs under hypoxic condition. These results demonstrated that autophagy inhibition can attenuate the angiogenetic effect of VEGF in RVECs.

### Expression of VEGF in RVECs was not changed by autophagy inhibition

**Fig. 7:**
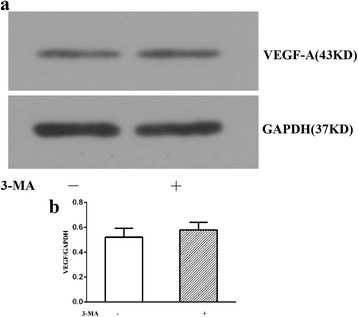
Representative immunoblot bands of VEGF of RVECs in the two groups (**a**), and statistical histogram of VEGF expression (**b**)

Western blot demonstrated that when pretreated with 3-MA under hypoxia, expression of VEGF-A of RVECs had no significant difference compared with the hypoxia group without 3-MA treatment (0.58 ± 0.06 vs0.52 ± 0.07, *P* > 0.05) (Fig. 7) (Additional file 2). This result indicated that inhibition of autophagy may exert no effects on the expression of VEGF in RVECs.

### Autophagy of RVECs was activated by anti-VEGF treatment

**Fig. 8:**
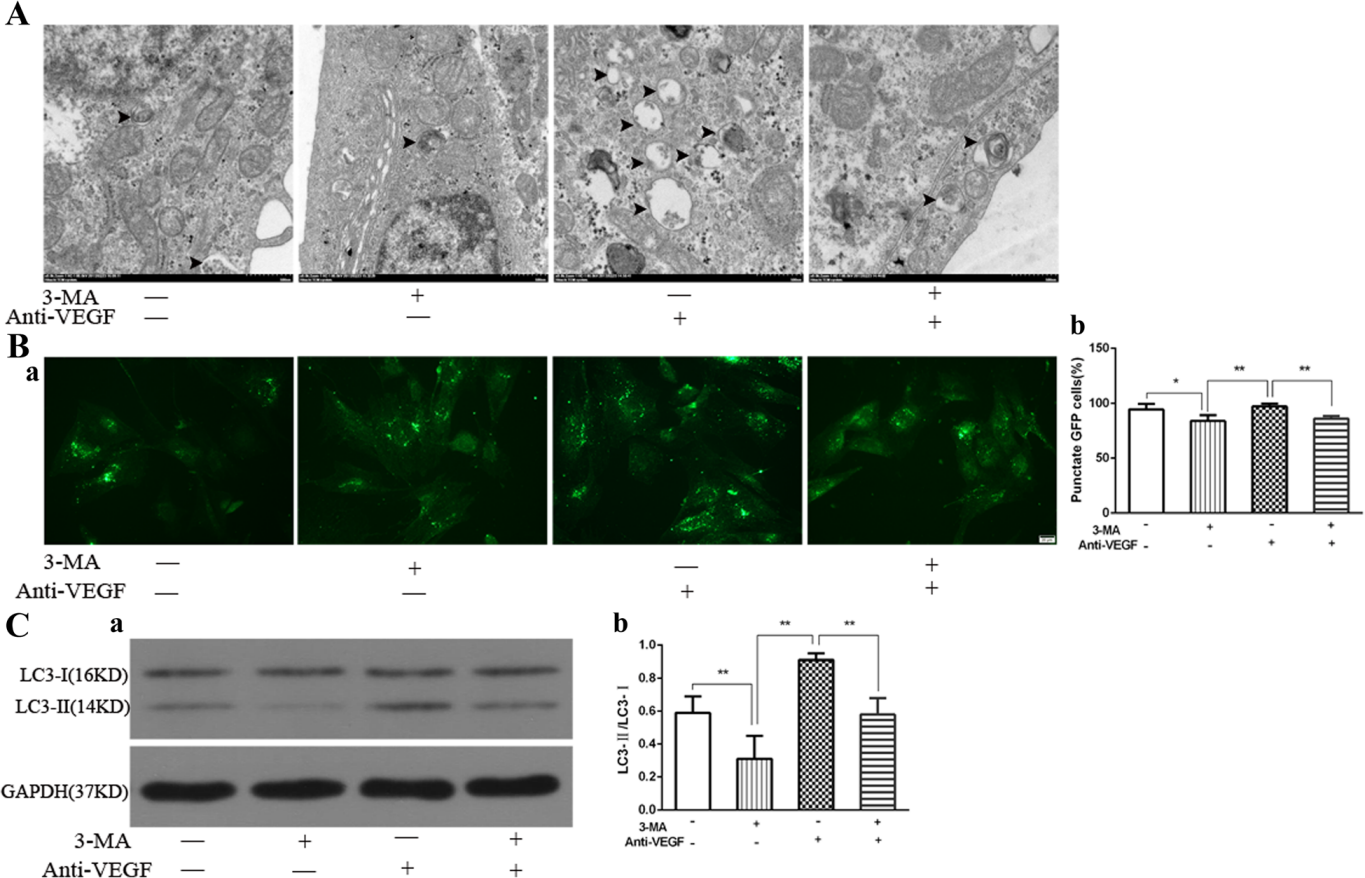
**a** Representative images of autophgosome formation in RVECs detected by TEM in the three groups (The arrow heads indicated the double-membraned vacuoles digesting organelles or cytosolic contents, Bar = 500 μm). **b** Representative images of endogenous GFP-LC3B visualized by fluorescence microscopy either as a diffuse cytoplasmic pool or as punctate structures that primarily represent autophagosomes (Bar = 20 μm) (a) and statistical histogram of percentage of punctate GFP cells in different groups (b) (*P<0.05, **P<0.01). **c** Representative immunoblot bands of LC3B-I, LC3B-II in RVECs in the three groups (a). The histograms were the quantification of 3 independent experiments on LC3B (b). The levels of these proteins were normalized to that of GAPDH (** *P*<0.01)

Formation of autophagosome was significantly increased in the anti-VEGF group compared with the control group and the 3-MA group and this enhancing effect of anti-VEGF antibody was blocked by pre-treatment with 3-MA (Fig. 8a). As TEM is a kind of qualitative detection method but not an ideal quantitative method to inspect the formation of autophagosome in cells due to the possible big errors in the process of sample preparation, only the qualitative results were presented here. In the anti-VEGF group, the number of GFP+ puncta was significantly higher than that of the control group (97.18 ± 2.50 vs. 94.44 ± 4.96, *P* < 0.05) and the 3-MA group (97.18 ± 2.50 vs. 83.98 ± 5.39, *P* < 0.01), and it was decreased in the 3-MA + anti-VEGF group (97.18 ± 2.50 vs. 86.00 ± 2.40, *P* < 0.01) (Fig. 8b) (Additional file 1). The protein expression level of LC3B-II/I in the control group, 3-MA group, anti-VEGF without 3-MA group and 3-MA plus anti-VEGF group were 0.59 ± 0.10, 0.31 ± 0.14, 0.91 ± 0.04 and 0.58 ± 0.10, respectively. Obviously, expression of LC3B-II/I was significantly higher in the anti-VEGF group than in the control group and the 3-MA group (*P* < 0.01), while pre-treatment with 3-MA significantly decreased it in RMECs exposed to anti-VEGF (*P* < 0.01) (Fig. 8c) (Additional file 2). In together, these results suggested that the expression of autophagic markers of RVECs was elevated in cells treated with anti-VEGF antibody and this effect could be partially inhibited by 3-MA pretreatment. In together, these data suggested that anti-VEGF antibody was a potent inducer of autophagy in RVECs under hypoxic condition and that the induction of autophagy by anti-VEGF could be partially blocked by 3-MA pretreatment.

## Discussion

Angiogenesis is a complex and multi-step process for the formation of new vessels from pre-existing capillaries. Pathological growth of new vessels, which is closely related to hypoxia, contributes to a variety of eye diseases, including retinopathy of prematurity, proliferative diabetic retinopathy and retinal venous occlusion [17–19]. A number of therapeutic strategies are being developed to inhibit pathological angiogenesis in recent years. VEGF is the strongest pro-angiogenic factor to induce proliferation of endothelial cells, promote cell migration, and induce budding of endothelial cells and formation of tubular structures [20]. Hypoxia and other cytokines can induce the expression of VEGF and intraocular levels of VEGF are elevated in patients with retinal or iris neovascularization, and VEGF-specific antagonists markedly suppress retinal neovascularization in mice and primates with ischemic retinopathy [21]. Therefore, VEGF has become a key target for the clinical treatment of pathological angiogenesis. However, the upregulation of VEGF is prolonged in many patients and no treatment can reliably reduce the production of VEGF, so repeated intravitreous injections of anti-VEGF drugs are commonly required and not all patients can be treated effectively [21]. This clinical phenomenon suggests that other important mechanisms are also involved in retinal angiogenesis.

Autophagy widely exists in eukaryotic cells, ranging from yeast to mammals, and plays essential roles in many physiological and pathological processes in living organisms. TEM is considered as the gold standard for qualitative detection of autophagy because of its direct presentation of the morphology of autophagosomes [15]. The proteins encoded by specific genes related to autophagy (Atg) were involved in various stages of autophagy formation in a coordinated manner. LC3, the homologous protein of Atg8 in mammalian cells, exists in both LC3-I (the cytoplasmic form of LC3) and LC3-II (the membrane-type of LC3). When autophagy occurs, LC3-I was converted to LC3-II by enzymolysis of a small amount of polypeptide [15]. Furthermore, LC3-II can be combined with GFP to form GFP-LC3 chimeric protein and detecting its content (GFP+ puncta/ total GFP-positive cells) can reflect the activity of autophagy [22]. As a subtype of LC3, LC3B is most commonly used as the molecular indicator of autophagy. The ratio of LC3B-II/I can be detected by western blot and the number of GFP-LC3B by immunofluorescent assay. These three techniques are often combined to inspect autophagy in order to make up for the defect of a single method. Under normal condition, autophagy maintains at a basic level. A large number of studies have shown that autophagy is activated under the conditions of starvation or hypoxia-ischemia to promote cell survival [11, 23–25]. However, over-activation of autophagy may also cause cell death [26]. We have previously shown that autophagy could be activated by chemical hypoxia (CoCl_2_) in RF/6A cells. In consistence, in the present study we found the activation of autophagy in RVECs under physical hypoxia, which is better mimicking the process of ischemic retinal diseases. In cells exposed to hypoxia, formation of autophagic vacuoles was significantly increased, the number of GFP+ puncta was also highly up-regulated and the ratio of LC3B-II/I was also obviously elevated compared to the control cells. In addition, activation of autophagy in RVECs induced by hypoxia was partially inhibited by pretreatment with 3-MA, which is a widely used cell-permeable autophagic sequestration inhibitor based on its inhibitory effect on phosphoinositide 3-kinase (PI3K) activity, which is known to be essential for induction of autophagy [27]. The molecular mechanism of autophagy is very complex and multiple signals may be involved, so autophagy was not completely blocked by 3-MA which inhibits PI3K signaling. When pretreated with 3-MA, the inductive effect of hypoxia on autophagy of RVECs was partially blocked. Besides, autophagy level in the hypoxia+ 3-MA group was still higher than that of the control group because autophagy maintained at a basic level for the controls. As 3-MA was reported to function only when activation of cell autophagy occurs, the 3-MA alone group was not set for detecting autophagy activation and cell migration and tube formation in RVECs under hypoxia [28–30].

In recent years, the relationship of autophagy with angiogenesis has been reported. Researches on tumor angiogenesis and cardiovascular diseases manifested that autophagy activation could promote angiogenesis [31, 32]. In nutrient deprived bovine aortic endothelial cells (BAECs), angiogenesis was reduced by inhibition of autophagy and migration and tube formation of BAECs was increased by induction of autophagy. In addition, inhibiting autophagy could impair vascular endothelial growth factor (VEGF)-induced angiogenesis [32]. In retinal angiogenesis, RF/6A cells in high glucose condition showed significantly increased expression of autophagy-related proteins, cell migration and tube formation compared to cells in low glucose and control conditions, suggesting that autophagy might participate in retinal neovascularization induced by high glucose [30]. In consistent, our previous study found that induction of autophagy under chemical hypoxia condition led to increased migration and tube formation in RF/6A cells and inhibition of autophagy by 3-MA suppressed angiogenesis [14]. In the present study, we further demonstrated that induction of autophagy in RVECs under physical hypoxia was associated with enhanced cell migration and capillary formation. Besides, angiogenesis of RVECs was weakened in the hypoxia+ 3-MA group when compared with the control group, in which cell migration and capillary formation were maintained at a certain level under normoxia conditions. Although autophagy level was higher in the hypoxia+ 3-MA group than the control group at 24 h, the process of angiogenesis was initially inhibited by 3-MA pretreatment. In addition, we found that VEGF-induced cell migration of RVECs was obviously attenuated by an autophagy inhibitor 3-MA by the combination of wound healing assay and transwell assay. The capillary formation assay demonstrated that exposure to VEGF treatment significantly increased the total tube length and the number of branch points, demonstrating that VEGF-induced tube formation of RVECs was also attenuated by autophagy inhibition. Furthermore, western blot demonstrated that expression of VEGF-A in RVECs did not change with or without 3-MA pre-treatment. Our results were quite similar to that of Du et al. [32]. All these findings suggested the role(s) of autophagy in retinal angiogenesis and that manipulation of autophagy might represent a novel therapeutic strategy in retinal neovascularization.

Then, we found that formation of autophagosome observed by TEM, the number of autophagosome in RVECs represented by GFP+ puncta under the fluorescence microscope and the LC3B turnover of RVECs tested by western blot were all significantly increased in the anti-VEGF + hypoxia group compared to the hypoxia group and this enhancing effect of anti-VEGF antibody was blocked by pre-treatment with 3-MA. In together, these data suggested that autophagy of RVECs was activated by anti-VEGF treatment, which was consistent with other studies in which angiogenesis inhibitors could induce autophagy [31, 33, 34]. These findings suggest that anti-VEGF agents may activate autophagy, which will then promote angiogenesis to finally attenuate the treatment effect of anti-VEGF on retinal neovascularization. This may partly explains why anti-VEGF therapy is not always effective for retinal neovascularization.

Hypoxia is recognized as the most important trigger for the process of angiogenesis and this study identified inhibition of autophagy of RVECs induced by hypoxia can attenuate the angiogenic effect of VEGF but not interfere with the expression of VEGF in RVECs. Autophagy of RVECs can be activated by anti-VEGF treatment. At present, no completely safe and long-term effective treatment for retinal neovascularization is available. Therefore, our findings may identify autophagic response as a novel target for enhancing the therapeutic efficacy of angiogenesis inhibitors. However, these preliminary in vitro findings may not completely represent their actual presentations in retinal neovascularization in vivo. And whether the clinically used drugs to inhibit VEGF have the same effects as the VEGF antibody used in this study is also unknown. Thus, the relationship between autophagy and retinal angiogenesis should be further explored.

## Conclusions

Oure present data may identify the autophagic response as a novel target for enhancing the therapeutic efficacy of angiogenesis inhibitors.

## Additional files

Additional file 1: Data of GFP-LC3B. The raw materials for statistics of percentage of punctate GFP cells in different groups. (XLS 84 kb)

Additional file 2: Data of western blot analysis. The raw materials of western blot of VEGF and LC3 in different groups. (XLS 1095 kb)

Additional file 3: Data of wound healing assay, transwell assay and endothelial cell capillary formation assay. The raw materials for calculating the wound healing rate, number of migrated cells, number of branching points and total tube length in different groups. (XLSX 15 kb)

## Abbreviations

3-MA: 3-methyladenine
RVEC: Retinal vascular endothelial cell
TEM: Transmission electron microscopy
VEGF: Vascular endothelial growth factor
